# The SIA Can’t Just Go with the FLO

**DOI:** 10.1007/s10730-023-09510-5

**Published:** 2023-07-13

**Authors:** Joe Slater

**Affiliations:** Philosophy Department, 67-69 Oakfield Avenue, G12 8LP Glasgow, UK

**Keywords:** Abortion, Ethics

## Abstract

Hendricks (2018) has defended an argument that abortion is (usually) immoral, which he calls the impairment argument. This argument purports to apply regardless of the moral status of the fetus. It has recently been bolstered by several amendments from Blackshaw and Hendricks (2021a; 2021b). In this paper, three problems are presented for their Strengthened Impairment Argument (SIA). In the first, it is observed that even with the new modifications the argument, contrary to their insistence, does seem to depend on Marquis’ argument. In order for it not to do so, they would need to provide some other plausible reason why impairing a fetus is wrong that persists in cases of abortion. Because of the restrictions regarding what reasons can be used, they are not entitled to stipulate that some plausible reason can be found. In the second section, the use of an over-ridingness caveat – the most recent modification – is scrutinised. This is shown to either beg the question about the permissibility of abortion by assuming that opposing reasons are insufficient in most cases, or require an entirely separate argument to establish that such reasons are insufficient. Thirdly, I observe that the principle utilised in the latest version of the argument fails to account for undercutting reason, which suggest that the principle, in its current form, is false.

## Introduction

The Impairment Argument, given by Hendricks (2018) and recently defended by Hendricks and Blackshaw in several co-written papers (2021a; 2021b), has received significant attention. The stated goal of this argument is to show that abortion is immoral (in almost all cases), and that this is true regardless of whether the fetus is a person. Whether the fetus is a person has been a hotly contested question and has often been seen to determine whether abortion is morally permissible,^1^ so arguments that can bypass that issue can be significant contributions to the abortion debate. This strategy – ignoring the question of personhood – is utilised in some of the most important contributions regarding abortion, including Thomson’s (1971) and Marquis’ (1989).^2^ The Impairment Argument is an indirect one. It starts with intuitions about someone knowingly drinking during a pregnancy and giving the fetus Fetal Alcohol Syndrome (FAS), a condition that can impose serious mental and physical disabilities. It then relies on a pivotal claim: if we think impairing the organism (the fetus) *that* much is immoral then, ceteris paribus, impairing it *more* must also be immoral. An abortion *must* constitute a greater impairment, as a successful abortion impairs the fetus maximally. Thus, the argument suggests, abortions are immoral in almost all cases.

The crucial move in Hendricks’ original argument is termed the Impairment Principle:

The Impairment Principle (TIP): if it is immoral to impair an organism O to the nth degree, then, ceteris paribus, it is immoral to impair O to the n + 1 degree.

Utilising TIP, the argument generated then looks like this:^3^

1. If it is immoral to impair the fetus by giving it FAS, then, *ceteris paribus*, it is immoral to kill the fetus.
2. It is immoral to impair the fetus by giving it FAS.
3. *Ceteris paribus*, it is immoral to kill the fetus.
4. To abort a fetus is (in most cases) to kill it.
5. So, *ceteris paribus*, to abort a fetus is (in most cases) immoral.

Following various criticisms, the argument has undergone several revisions. Recent versions of the argument have dispensed with TIP, because the ceteris paribus caveat is not met when moving from the FAS case to abortion, as pointed out by Crummett (2020) (and acknowledged by Blackshaw and Hendricks (2021b). Its successor, the Modified Impairment Principle (MIP), which was vulnerable to a series of counterexamples (acknowledged in Blackshaw and Hendricks, 2021a), has also been abandoned. Instead, Hendricks and Blackshaw currently defend MIP2:

MIP2: If it is immoral to impair an organism O to the nth degree for reason R, then, provided R continues to hold (or is present) *and there are no over-riding reasons*, it is immoral to impair O to the n + 1 degree (Blackshaw and Hendricks 2021a).

MIP2 differs from MIP only in the italicised caveat regarding over-riding reasons. The modifications were required to avoid the implication that causing an impairment is wrong when doing so looks commendable. Consider two cases:

### Villain

A villain stabs you, at a whim, puncturing your left kidney, leaving that kidney impaired but still functional.

### Surgery

At your request a surgeon performs a nephrectomy, and transplants one of your kidneys into a needy patient. You are left with one functioning kidney, and with kidney function impaired to a greater degree that in Villain.

The original MIP implies that the surgeon would act immorally in Surgery. Under MIP2, this problem does not obtain, because the saving of the recipient’s life is clearly an over-riding reason, which can justify the surgery.

The following is Blackshaw and Hendricks’ characterisation of their argument (2021b), with MIP2 replacing the ceteris paribus clause, i.e., the most recently defended version of their argument, the Strengthened Impairment Argument (SIA):

1. If it is immoral to impair the fetus by giving it FAS for reason R, then, so long as R applies *and there are no over-riding reasons*, it is immoral to kill the fetus.
2. It is immoral to impair the fetus by giving it FAS, for R.
3. If R applies, *and there are no over-riding reasons*, it is immoral to kill the fetus.
4. To abort a fetus is (in most cases) to kill it.
5. So, to abort a fetus is (in most cases) immoral, if *there are no over-riding reasons* and R applies.

In order for this argument to actually give an argument against abortion, some R is required such that it is a reason why it is at least plausible that it is impermissible to give a fetus FAS, *and* that it is still applicable in abortion cases. Thus we may supplement this with the following:

6. There is a reason, R, which is why it is immoral to impair a fetus by giving it FAS, *and* which applies in (most) abortion cases.
7. To abort a fetus is (in most cases) immoral, *if there are no over-riding reasons*.

(1) is a derivation from MIP2. (4) is definitional. (3), (5) and (7) are valid deductive inferences, if some R is given such that it satisfies (2) and (6). Blackshaw and Hendricks have suggested (2021b) that a reason that is suitable for these purposes is given by Marquis (1989). Marquis claims that the (primary) reason it is wrong to kill an adult person is that it deprives them of a Future Like Ours (an FLO). This has formed the one of the most famous anti-abortion arguments in recent decades,^4^ and received considerable attention.

In Marquis’ argument from “Why Abortion is Immoral” (1989), he claims that what makes killing fetuses wrong is the same as what makes killing an innocent adult human wrong. This, he claims is an FLO. An FLO may contain all sorts of things that make one’s life good. It is a “future of value” (2007, 399). For some, a devoted family life may be crucial, for others a career or sports may take priority. For adult humans, a multitude of features will probably give their lives value. What makes it wrong to kill an innocent adult human then is that it deprives them of these features. Marquis argues that the same applies to fetuses – they have an FLO, so this makes it severely wrong to kill them.

An advantage of Marquis’ account is that it is able to give a nice explanation of why causing death is wrong, when it is in fact wrong, and also provides the resources to say that some instances of killing are not in fact wrong. If someone has a terminal illness and is suffering, they may not have an FLO, so euthanasia, even active euthanasia, may be permissible (Marquis, 1989, 191). Similarly, if someone falls into a coma and we know they will never wake up, allowing them to die does not deprive anyone of an FLO. Despite the advantages of Marquis’ account, it has attracted many criticisms,^5^ but these are not relevant for my present purposes.

In the next three sections, I demonstrate three problems for the SIA. The first concerns giving a suitable R, such that the argument isn’t simply a restatement of Marquis. For the argument not to be redundant, there must be some reasons – other than deprivation of FLOs – that can be implanted into the argument. However, because of constraints imposed by the goals of the argument, this is not as easy as it looks. From a short consideration of prima facie plausible candidates, I argue that this is a significant challenge, so the existence of such reasons cannot be taken for granted. I regard this as a challenge for the SIA, but not a fatal problem, unlike the following which I regard as more damaging. The second is that the *over-riding reasons* caveat included in MIP2 cannot succeed in establishing the desired conclusion unless accompanied by additional value judgments, which the argument’s opponents will not share. Thus, their claim that R is not over-ridden in most instances of abortion is left either begging the question or in need of some argument to establish that the reasons against abortion are sufficiently weighty (i.e., they need an argument against the permissibility of abortion!). Third, MIP2 makes no allowance for undercutting reasons, which can detract from the force of a reason. Because of these issues, I argue that the SIA is unsuccessful.

## Reasons Other Than FLO?

Gillham has argued that the SIA simply restates Marquis’ argument (2020; 2021). He observes that Hendricks and Blackshaw do “not make it entirely clear why they take SIA and Marquis’ account to be different” (2021, 839). There is a conceptual confusion here that can be clarified.

Marquis gives an account of *what makes* certain actions wrong. His FLO account says that killing is wrong (when it is wrong) primarily because it deprives someone of a future like ours. He does concede that there may be other additional reasons that make killing morally objectionable, but sees deprivation of an FLO as the most important reason in most cases. He does not elaborate exactly what features of an FLO make the deprivation of them bad (which makes his account compatible with different accounts of wellbeing), but that an act deprives a subject of them counts morally against it. Because of this, Marquis claims, only very weighty considerations can justify these actions, such as when another person’s FLO is at risk, e.g., self-defence cases or when a pregnancy could prove fatal for the pregnant party.

The FLO account offers an explanation for the wrongness of certain actions (mostly actions involving killing) and applies this to cases of abortion. The SIA, however, gives us a formula or blueprint. If the argument’s formula is successful, then once some suitable reason, R, is plugged in, it demonstrates that abortion is wrong. The only such candidate reason mentioned by Blackshaw and Hendricks is the one Marquis gives; that depriving an individual of an FLO is wrong (except in extreme circumstances, e.g., self-defence). Once this reason is given, they have a valid argument. However, Gillham has argued that if revised this way, they merely restate Marquis, thus defeat the point of the Impairment Argument.

Contra Gillham, Blackshaw has recently pointed out that “the SIA is not committed to deprivation of FLO as the sole reason this impairment is immoral, and so it is not an essential part of the argument” (2021, 841). Because other wrong-making conditions – other candidates for R – could be utilised in the SIA it is not *in principle* beholden to Marquis’ argument. And Blackshaw seems hopeful regarding those prospects, claiming “there may be a superior explanation we have not considered” (2021, 841).^6^

If there is no suitable candidate for R other than deprivation of an FLO, the SIA is redundant. To avoid this fate, SIA must hold the following:

6*: There is a reason, R, *other than deprivation of an FLO*, which is why it is immoral to impair a fetus by giving it FAS, *and* which applies in (most) abortion cases.

The problem for SIA is that it is very hard to see what other explanations might serve their purposes.

Due to the goals of their argument, alternative Rs must have the following features:

A. It must not rely on claims that a fetus is a person.
B. It must be present, and constitute a bad-making feature in the FAS case.
C. It must be present in the abortion case.

(A) is required because one of the stated goals of the argument is to circumvent discussions of personhood. (B) and (C) are needed so the impairment can be shown to be bad in the “n” case (FAS), and that MIP2 can be used to generate a conclusion for the “n + 1” case (abortion).

If the argument is to be persuasive for people who currently view abortion as permissible, the reasons provided must also be available to them, as something they would recognise as a reason. I take this to add a further restriction.

D. It must not rely upon controversial ontological claims.

If a reason is given that meets (A)-(C), but not (D), this could allow the argument to be non-redundant. However, the argument would be of diminished utility, and, as far as people who reject the ontological commitments go, it would still add nothing to Marquis’ argument. I return to this point shortly.

Conditions (A)-(C) may not seem so onerous, but they are very restrictive. Consider some of the possible reasons why it is wrong to cause a fetus to have FAS, i.e., reasons which meet condition (B). For example, one might consider a utilitarian answer. Causing a fetus to have FAS will likely cause a reduction in expected utility; the resulting child may have serious physical and cognitive disabilities that could reduce their wellbeing. At first glance, one might think that considerations of expected utility are also a reason against having an abortion, so it should satisfy condition (C). This might look plausible, because even if the pregnant party would prefer an abortion, not having one will result in a human being born, with the potential for a rich, enjoyable life, i.e., one that would bring about a lot of additional utility. And, of course, one doesn’t have to be a utilitarian to think that the amount of happiness is morally relevant. So, we might suppose that this would make a nice candidate for R.

But this is too quick. While it is a very difficult empirical question to answer whether any given abortion (or lack of) actually results in an increase or decrease of utility, as we can never witness the counterfactual, there are a host of considerations that make it is very plausible that in some cases an abortion taking place will increase the expected utility. If someone was prevented from obtaining an abortion, despite not wanting the pregnancy, this could be psychologically damaging. It could have pernicious consequences on their self-worth. This may affect how they parent, which could in turn have detrimental effects on the child. Additionally, some who become pregnant may intend to instead have a child in different life circumstances, such that they would be better prepared for parenthood, emotionally and financially. If it is expected that they would be successful in this goal, having an abortion now might improve their own quality of life significantly, and help to bring about a child with improved wellbeing.^7^ Given these considerations, I suggest that we should accept that abortions are sometimes for the best, in terms of general utility. And if we accept that, this candidate reason why imposing FAS is wrong (i.e., that it reduces expected utility) fails condition (C). Someone who attempted to use this as an R would only be able to establish that abortion is wrong when the expected utility of carrying the fetus to term was higher. This could be used to argue for impermissibility of abortion on a case-by-case basis – assessing the expected utility in every case and making a determination about expected utility – but could not yield a conclusion nearly as strong as Hendricks and Blackshaw aim to provide.^8^

Another plausible reason that intentionally inflicting FAS on a fetus is bad is that it harms its future interests. The suggestion that what makes prenatal harm morally bad is that the organism *will have* certain interests – interests which are impeded by the prenatal harm – is defended by Steinbock (1992). The fetus that goes on to be born with FAS will have interests in being physically and cognitively able to function; these interests are hindered by their having FAS. This reason meets condition (A), as it does not rely on the claim that the fetus is a person; it only requires that it *will* have interests. But this fails to meet condition (C), as the aborted fetus will never grow to have those interests.

A slightly different route would be simply to consider *interests*. It might be suggested that the fetus already has some interests, like an interest in continued survival. This would not entail anything about its personhood-status. Defenders of the interest theory of rights, for instance, often speak about non-persons (like young infants or animals) having interests, e.g., Kramer (1998). However, this is unsuitable for use with SIA for several reasons. First, it is unclear that this reason applies in the FAS case. While we might claim that a fetus has an interest in *surviving*, it does not yet have the capacities to have many of the interests that persons have. A fetus may not have an interest in mobility or cognitive development, simply because it is not yet the kind of being that can have such interests. For this reason, to apply in the FAS example, a case needs to be made for a fetus already having interests which are thwarted by FAS. Perhaps a successful argument for this can be made, which demonstrates that a fetus does have interests, but a further problem awaits. If the harm to its interests is thought to make impairing a fetus morally wrong, it looks like the argument overgeneralises, as flies or even parasites are likely to have similar interests. Because of the overgeneralisation challenge, the harming of interests does not look like a promising candidate for R.

It is open to a virtue ethicist to locate reasons in what the action shows about the person who has an abortion. They can provide character traits that they identify as virtues and suggest that someone knowingly inflicting FAS on a fetus exhibits them. Perhaps they act callously, or irresponsibly. However, it is less clear that these traits are typically manifested in the abortion case. Hursthouse, for instance, suggests that someone *can* exhibit these vices in seeking an abortion, but often does not (1991). While inflicting FAS might be appropriately called irresponsible, this label doesn’t fit so obviously on someone who became pregnant due to a failure of contraception, and wants to obtain an abortion because being pregnant now does not fit within their life plan. So, (C) may not obtain. Furthermore, even whether we view the person as callous (or some other vice) in the abortion case might depend on whether we regard the fetus as a person, so (A) could also fail.

Yet another route would be to consider a natural law view. As this approach has a history of being deployed in anti-abortion arguments,^9^ this could look promising. Here the candidate R would be that some natural law or natural right is violated. Finnis, for example, argues that we must be “adequately open to, attentive to, respectful of, and willing to pursue human good insofar as it can be realized and respected in our choices and dispositions” (1973: 126), and that this generates a right not to be killed intentionally. This would be a right grounded in human nature, which a fetus would possess in virtue of being a human organism. A related move comes from substance view theorists, who argue that because a fetus is constituted of the same kind of substance as us, a rational substance which has inherent moral worth.^10^

Attempts to provide some R along either of these lines might have the best prospects for success of those mentioned so far. However,^11^ these approaches violate – or look *very close* to violating – the (A) condition. Substance theorists often describe their view as the substance view *of persons*,^12^ making this assumption explicit. Natural law theorists do not need to talk in these terms;^13^ they could try to make the case that the basic value of human good can be disentangled from what it is to be a person, such that it does not entail a position holding that the fetus is a person. Perhaps either of these views can make the case without illicitly (for the purposes of SIA) smuggling in the (A) condition, but again, this is not an easy prospect.

It is worth pointing out that the whole class of reasons given by person-affecting views of morality will be excluded by condition (A). These are views that hold that no action is wrong unless there is someone – some *person* – who is wronged by the action.^14^ This rules out any reasons given by several popular views of morality. For instance, Scanlon’s contractualism (1998) is explicitly person-affecting. Typical forms of contractarianism will be too. On some interpretations, so is Kant’s moral theory.^15^ Others, like Heyd explicitly endorse the person-affecting approach (2009). But because person-affecting views require, in order for a wrong to be done, “a person’s having been made *worse off*, or *harmed*, or *wronged*” (Roberts & Wasserman, 2009, xiv), no person-affecting view can be suitable for the SIA.

One option I have not mentioned is one that I suspect moves many people in discussions of abortion, namely, that the fetus has a soul. If one accepts certain theological commitments, this could meet conditions (A)-(C). That the fetus has a soul could provide a reason not to impair it by imposing FAS on it. If ensoulment occurs at conception, this reason would still be present for any given instance of abortion. And, we might say that an ensouled being is not (yet) a person, so (A) could be met. The main issue with this reason is that it would fail to satisfy condition (D), so render the argument entirely unpersuasive to those who do not accept those commitments, i.e., to those who do not believe in souls. It would also not justify the SIA’s conclusion for any theists who believe, like Aristotle, that ensoulment occurs later in a pregnancy. Furthermore, many who do believe that ensoulment occurs at conception will also already accept the conclusion. Despite these issues, this reason – if filled in with appropriate details – *could* fit into the SIA. However, due to the controversial ontological commitments, this may be independently undesirable.

Other candidates for R that could be offered which violate (D) include papal decree, moral intuitionism, or divine command theory. Such options show little promise of persuading anyone who doesn’t already harbour an anti-abortion stance. Furthermore, for most (or all) of these options, the SIA seems redundant, because it is entirely unnecessary. If, for instance, someone believes that God commands them not to harm fetuses, and that this explains the wrongness of imposing FAS on a fetus and of abortion, the SIA is not needed. These background beliefs are sufficient for deriving the conclusion, without use of the indirect impairment argument.

Other reasons *could* be offered for R, perhaps even that meet condition (D), but the goals of the argument are severely restrictive. It seems striking that the only R offered is one that has already been deployed in a popular anti-abortion argument (Marquis’). The project of offering a plausible candidate is difficult because most of the standard reasons we would give to explain why it is wrong to cause an individual to be cognitively and physically impaired do not apply to a fetus. If Blackshaw and Hendricks want to decouple the SIA from Marquis’ argument, they must offer a plausible alternative R. Otherwise, it remains dependent on Marquis’ argument, and a worse version of it because it requires other intuitions (about FAS and MIP2); the argument would be redundant, at least in the eyes of those who disagree with the controversial metaphysical claims.

Alternatively, they may stipulate 6* – recall:

6*: There is a reason, R, *other than deprivation of an FLO*, which is why it is immoral to impair a fetus by giving it FAS, *and* which applies in (most) abortion cases.

Making 6* explicit highlights a problem. Simply claiming that there *is* some such an R begs the question against opponents. A brief consideration of the most obvious candidates, as provided above, demonstrates that there is a serious difficulty in finding an R that appears satisfactory. If any R that is offered violates (D), the argument is rendered unpersuasive, absent arguments for the controversial metaphysical position. Furthermore, even if it is acknowledged that Rs can be given that violate (D), as well as these being unpersuasive for opponents, the SIA still faces the objections I provide in Sects. 3 and 4.

The preceding objection to SIA is not a fatal one. It is merely intended to shift the burden onto anyone who wants to defend the argument. The following argument, however, I do take to pose a significant problem, even if some satisfactory R could be offered.

## Over-Riding Reasons?

Let us return to MIP2, which is a crucial part of the SIA:

MIP2: If it is immoral to impair an organism O to the nth degree for reason R, then, provided R continues to hold (or is present) *and there are no over-riding reasons*, it is immoral to impair O to the n + 1 degree.

As I stated earlier, the modification of MIP to MIP2 avoids some of the counterexamples. Recall “Surgery” and “Villain”. In “Surgery”, impairing someone by removing a kidney (with consent) so that it may be transplanted into someone needy offers a justification that is not present in “Villain”, where a person is stabbed in same kidney out of sadistic pleasure, even though the former case may actually result in a greater impairment. So, as Blackshaw and Hendricks claim (2021a), this revision does avoid the counterexamples proposed by Crummett (2021).

When this is applied to the abortion case, however, things become less clear. In “Surgery”, we have very clear intuitions that this is permissible. Features of the case – like the great improvement for quality of life for the recipient and the consent of the donor – do give over-riding reasons, so the impairment is permissible. But, in more controversial cases, determining what reasons are over-riding is not such an easy matter.

Blackshaw and Hendricks describe their over-rider provision like so:

“[A] reason R only renders an impairment immoral if it is not over-ridden by an opposing reason R*” (Blackshaw and Hendricks 2021a).

In Surgery, R* might be the combination of the informed consent from the donor^16^ and the expected benefit to (and consent from) the recipient (I take these to be jointly sufficient for the surgery being permissible). In “Villain”, no reason is satisfactory for R*. The villain may protest that the sadistic pleasure they gain should over-ride the reasons for refraining from the harm, but we would apply *value judgements* to the situation, and dismiss such a plea without hesitation. This is crucial. For the argument to be successful – even in the cases of “Surgery” and “Villain” – we need to consult an existing standard for how valuable the reason for causing the impairment is, *and* how disvaluable the impairment is. In “Villain”, the impairment can be condemned for many reasons, which we consider to be very weighty, and we judge that any pleasure gained from the villain from their evildoing doesn’t come close to over-riding those reasons. In “Surgery”, the impairment is worse, but we take the opposing reasons (the donor’s consent and the expected benefit to the recipient) to be extremely strong. Without these independent judgments, the argument gets us nowhere.

Now consider two impairment cases relevant for the SIA:

### Excessive Pregnancy Drinking

A pregnant person, knowing that they are pregnant and knowing the risks of drinking, gets regularly drunk during their pregnancy. In doing so, they give their fetus FAS.

### First Trimester Abortion

Following an accidental pregnancy, the pregnant party weighs their options, considering the mental, physical and financial costs. Ultimately, they decide to have an abortion, which kills the fetus.

There may be several different reasons that might make us condemn the actions in “Excessive Pregnancy Drinking”, e.g., that the behaviour causes harm to a fetus, that it displays a disrespectful attitude,^17^ that it deprives the fetus of an FLO, etc. These would be the R we can place into MIP2. Perhaps, in most cases, there are no reasons they could give would convince us that these actions are permissible. (This is clearly what Blackshaw and Hendricks accept; I will take no position on this here.) If that is the case, no suitable reasons could over-ride the R, i.e., there could be no R*.

Yet in “First Trimester Abortion”, there are many things that can be said as a defence. Taking a pregnancy to term is a life-changing experience and one that incurs serious physical and emotional burdens, as well as a risk of death. If one intends to raise the child, this means a huge sacrifice of one’s time, energy and money. And even if they gave the child up for adoption, the emotional toll of that ordeal should not be underestimated. This option is often suggested by those arguing against abortion (e.g., Hendricks, 2018: 250) as though it is an easy option, but this is clearly not the case.^18^ Even putting to one side the serious psychological costs that may be involved in taking a pregnancy to term, the physical demands of pregnancy are robust. Because of these considerations there are substantial benefits obtained by abortions.^19^ The avoidance of each of these could serve as a candidate for R*.

When this is all exposed, whether these reasons are over-riding or not once again comes down to applying a value judgement. Moreover, it depends *what* the R is. To insist that any opposing reasons are not over-riding, in absence of knowing what they are in contention with, is to beg the question. Whether the reasons do over-ride any negative features of abortions is precisely what is at stake.

The argument fails because we must consult some independent standard to determine whether some R is over-ridden by some candidate R*, and the argument gives us no way of favoring one set of reasons or another. An obvious way to respond to this would be to supplement the argument with some additional mechanisms to determine which reasons over-ride which other reasons. However, if Blackshaw and Hendricks get involved in arguing *why* the moral features favour one side or the other, they will need a new argument, as nothing in the SIA provides the tools for evaluating value differences. If this strategy is taken, the SIA is once again redundant. Arriving at a way to appropriately compare relevant values is a mammoth task, and may not even be possible.^20^ So even updated with MIP2, and even if a suitable reason is given (that meets the criteria discussed in the previous section), the SIA is unfit for purpose.

We might suppose that, if a suitable R could be given, that this will help provide the justification for ascertaining whether some opposing reason can over-ride it. For instance, if we accepted that the deprivation of an FLO is an extremely grave moral wrong (a la Marquis), such that it explains why killing is wrong (when it is wrong), this explanation can be considered when thinking about what could over-ride it. Marquis suggests, for instance, that killing in self-defence can be justified because one person’s FLO is at stake (Marquis, 2014, 145) – an FLO justifies the restriction, but another FLO is weighty enough to over-ride it. Again, however, we see that all the work is being done by the R given (the FLO account), rather than the SIA.

### A Response: Misconstruing the Argument?

One might reply that this misconstrues Blackshaw and Hendricks’ intentions when formulating the SIA.^21^ Rather than demonstrating the wrongness of abortion, we might take the SIA is instead taken to show that aborting a fetus is a very morally weighty decision, and that a very good reason is needed in order to render this permissible. This could then be utilised to show that there is something wrong with what Driver (1992) calls “frivolous abortions”^22^ such as someone procuring a late-term abortion in order to go on vacation at a more convenient time.

In response, first, I disagree that this what Blackshaw and Hendricks intend to show. They have stated their conclusion as “abortion is immoral” (Hendricks, 2018; Blackshaw and Hendricks, 2021a, b), “abortion remains immoral” (Hendricks, 2019) and establishing “the immorality of abortion” (Blackshaw, 2021). I do not see any indication that this is supposed to show that is immoral *unless there is a very good reason*. Of course, it is open to them to weaken their conclusion to the weaker claim, but even this will not work.

Second, even if this is what the argument is intended to do, it fails in this task. While I agree that this *is* a weighty decision, the SIA does not possess the means to demonstrate this, because we can run a low stakes version of the argument. Consider the following toy example. Say that impairing a tree – e.g., cutting off most of its branches – for absolutely no reason would be wrong.^23^ Let this be the n-degree of impairment to stick in MIP2. Most reasons that apply to that would apply to killing the tree (the n + 1 degree). Yet it does not follow from this that an extremely weighty reason is needed to oppose destroying a tree. It still seems plausible that fairly minor desires could outweigh this. Perhaps someone wants to destroy the tree so they have a better view, for wood for a campfire, or to build a bench. These are not weighty reasons, but may be taken to over-ride the bad of killing a tree. So simply because we can run MIP2 says nothing about how weighty the moral wrong in question is, and consequently, says nothing about how weighty an opposing reason must be to over-ride it. For the SIA to be successful, prior judgements are needed about the moral value placed on the life (and death) of the fetus.

To be clear, one need not accept the judgments about damaging or killing a tree in order to accept my point. I simply intend to show that the ability to run the SIA (in any context) does not demonstrate that the over-riding reasons need to be very morally weighty. So long as we think it consistent to accept an instance where wantonly seriously damaging an organism (be it a tree, a flower or an insect) would be wrong, yet killing it for a non-weighty reason (making a bench, giving a flower to romantic partner, ceasing an annoying buzzing) would be permissible, we are committed to thinking that impairing to some n + 1th degree (killing) does not require a weighty over-riding reason. So, that the SIA can operate over a fetus in the n case (FAS) and the n + 1th case does not entail anything about how morally weighty an over-riding reason must be.

We might still wonder about the strength of the SIA; it is left somewhat ambiguous exactly how strong the conclusion of the SIA is intended to be. Hendricks doesn’t specify precisely which instances of abortion he takes to be immoral – and this is also unspecified in the co-authored papers from Blackshaw and Hendricks. From some of Blackshaw’s other works (e.g., Blackshaw and Rodger, 2020), it appears that Blackshaw accepts the permissibility of clinically indicated abortion, but perhaps few other cases.^24^ I take it as a fair reflection of their view that they see non-clinically indicated elective abortions as immoral. They do seem to embrace the conclusion abortion is immoral in most cases (Blackshaw & Hendricks, 2021a; Blackshaw and Hendricks, 2021b, 516).

Accepting this, not only the “frivolous abortions” mentioned by Driver, but also abortions favoured for socioeconomic reasons or a desire to limit childbearing, as these are, as Blackshaw and Rodger point out (2020, 179) are the most frequently reported motivations for abortions. What implications does this have for what may count as over-riding reasons? For the argument to deliver this conclusion – that abortion is immoral when the motivation is socioeconomic or because of preferences about the number of children – the strength of reasons offered by these motivations must be seen to be weaker than whatever R is offered for importing into the SIA.

Here, however, we return to the difficulty found above. Without knowing the R, we cannot know whether an R* including socioeconomic or family-size preferences over-rides it. And, even if we *did* know the R, this does not guarantee that there would be no disagreements on whether R or R* was morally weightier. So, we cannot know whether the “and there are no over-riding reasons*”* caveat of MIP2 obtains. Without that, no conclusion follows.

## Undercutting Reasons

Finally, one additional concern with MIP2 is that it ignores the possibility of undercutting reasons. A reason is *undercutting* if it when it obtains, it “makes it the case that what would otherwise be a reason is not a reason after all” (Schroeder, 2011), or in the case of *partial* undercutting reasons, makes the original reason less forceful. Depending on the R plugged into the SIA, it may be possible to give undercutting reasons. For instance, one might suggest that because it is wrong to prevent a human being flourishing (this does look independently objectionable as an R candidate, but bear with me for purposes of this illustration). While impairing a human being seems bad, impairing one that has never been conscious might (call this undercutting reason, R**), to many, seem less bad. So, this reason may still be *present*, but, given R**, may have little or no normative force. And such an undercutting reason – or the possibility of undercutting reasons – threatens MIP2, as a reason why one instance of impairing might be bad may still be present but undercut in the case of a greater impairment.

To provide another example, imagine we accept that one reason it would be bad to inflict FAS on a fetus would be to deprive it of the future ability to perform complex mathematical calculations.^25^ Specifically, I refer to the reason of it being bad *for the fetus*, not merely impersonally bad that there will be one fewer entity able to perform these tasks. This would certainly obtain in the abortion case – the fetus would definitely not be able to perform complex mathematical calculations later in life if it is aborted. But any normative force of this reason is undercut by the R** that the organism will not become a person. Non-persons have no need to use complex mathematical equations. So, while it is still *true* that aborting a fetus will prevent it later solving complex mathematical equations, this loses all its force as a reason against abortion.^26^

Exactly what will count as an undercutting reason will depend on what Rs are given. Regardless, these serve as putative counterexamples. If they are accepted, they demonstrate that MIP2 is false. It is of course open to Blackshaw and Hendricks to revise the principle again, as follows:

MIP3: If it is immoral to impair an organism O to the nth degree for reason R, then, provided R continues to hold (or is present) and there are no over-riding reasons *or undercutting reasons*, it is immoral to impair O to the n + 1 degree.

With this revision, the putative counterexamples no longer succeed. However, what I take to be the main problem from the previous section still remains. To determine which reasons are sufficient to over-ride, the defender of the SIA needs to bring in independent value judgments about the moral importance of killing a fetus. As these value judgements are not shared by their opponents, to claim that they are not over-ridden by the physical and mental costs of pregnancy (which is needed to establish the conclusion that abortion is in most cases wrong) is to assert precisely what is disputed, i.e., it begs the question. In addition, the same problem is now posed with regard to under-cutting reasons. Lacking knowledge of what the R is, we lack the context to know whether it has been undercut in the abortion case. Because of the possibility of undercutting reasons, MIP2 is false. Even if MIP3 were used instead, it is useless for establishing any claims about the permissibility of abortion, because without knowing what the R is, it is impossible to know in advance whether it is over-ridden or under-cut by other reasons present.

## Conclusion

The Impairment Argument has revitalised philosophical discussion about abortion. However, in its original form and in each of its modifications, significant problems have plagued the argument. Because of the problems pointed out here, the Strengthened Impairment Argument fails to offer any new reasons against the permissibility of abortion.

## Data Availability

There are no data in this work.

## Footnotes

1 E.g., Warren (1973); Li (1997); Harman (1999).

2 To clarify, when I mention “abortions” within this paper, I refer to medically induced abortions. These can be distinguished from “spontaneous abortions”, or miscarriages. For some of the discussion regarding the moral status of these, see Bohn (2021) or Blackshaw and Rodger (2019).

3 This version is given in Blackshaw and Hendricks (2021a, 2021b, 516). Hendricks again expresses the argument this way (2023).

4 Marquis’ argument has been described as the “best secular argument against abortion” (Strong, 2008).

5 E.g., that it faces an Epicurean challenge (Christensen, 2019), that it depends on animalism (Sauchelli, 2019), and that it implies that contraception is immoral (Norcross, 1990).

6 Previously, Blackshaw and Hendricks suggested that they *did* need to use deprivation of an FLO as a reason. They made this move because they wanted to condemn giving a fetus FAS even if that fetus is never born, and said “the only way to explain this fact is by appealing to the deprivation of the original FLO of the fetus; there are no other plausible explanations of its wrongness” (2021b, 517). Blackshaw and Hendricks seemingly had altered their position shortly afterwards, saying FLO was “merely as *one possible explanation* of the wrongness of impairment” (2021a, 641), and Blackshaw acknowledging the possibility of “a superior explanation” (2021, 841).

7 This is similar to Parfit’s example from *Reasons and Persons* (1984) of the 14-year-old who considers becoming pregnant.

8 I thank a reviewer for pressing me to express this point more precisely.

9 E.g., By Finnis (1973).

10 For recent discussion of substance view theory and abortion, see Simkulet (2019).

11 My purpose here is not really to challenge these views fully, but simply to assess whether they are suitable for the SIA. For some consideration of implications of these types of accounts, and criticism, see Lovering (2013).

12 E.g., Beckwith (2007).

13 Though they often do, e.g., Finnis (2007).

14 Many take the non-identity problem (Parfit, 1984) to be a fatal blow for person-affecting views, but they are still widely-accepted.

15 Mulgan (2006) discusses both interpretations of Kant that use the person-affecting approach and contractarian accounts.

16 As mentioned in (4), the donor’s consent is probably better viewed as an undercutting defeater, rather than an opposing reason.

17 For the reasons given in the previous section, I am sceptical about the prospects of these suggestions as candidates for R, but for the sake of this argument, we can assume that one of these succeeds.

18 For illustration, see discussion of grief by birth mothers by Krahn and Sullivan (2015).

19 Some of these benefits are discussed in Räsänen’s response to the original impairment argument (2020).

20 This is suggested by Pickard’s (2020) discussion of the argument.

21 I owe this response to an anonymous reviewer, as well as the clarification of what I regard Blackshaw and Hendricks’ conclusion to be, later in this section.

22 I am uneasy even mentioning this term, because the suggestion that people are regularly getting abortions “frivolously” seems absurd and objectionable. However, this is the extreme case of an abortion for no good reason, and I intend to show here that the SIA isn’t even able to condemn this category.

23 Perhaps because it is disrespectful to nature, or because there is *some* value in plant life, or because it removes a habitat for small animals. This seems like the sort of claim that Hendricks would accept, as he says it would be immoral to kill a tree for no good reason (2018, 252).

24 Blackshaw even argues that pro-life theorists should even reject the possibility of abortion in cases of rape (2022).

25 The claim that the resulting child would be *deprived* of this ability is probably too strong, as it is possible that someone with this condition may flourish despite the odds. If this is seen as an issue, the reason can be weakened to *severely hindering* this ability, or something similar.

26 A third example can be seen in the Surgery case above. The consent of the donor is best seen as an undercutting defeater, against the wrongness of violating someone’s bodily integrity. This is likely only a partial undercutter, as it would be immoral of a surgeon to perform an unnecessary nephrectomy (i.e., removing a kidney with no recipient in mind for no reason) even with a patient’s consent.

## Declarations

### Provenance and peer Review

Not commissioned; externally peer reviewed.
